# Supplementary material for: Genome-wide CRISPR Screens in T Helper Cells Reveal Pervasive Crosstalk between Activation and Differentiation
Source: Cell. 2019 Feb 7;176(4):882–896.e18. doi: 10.1016/j.cell.2018.11.044 (PMC6370901; doi:10.1016/j.cell.2018.11.044)
Supplement: Data S2. Processed Data from All the Steps of the Analysis, Related to Figure 1 [file mmc2.zip › supplemental data/motif analysis/Pparg_homer/homerResults/motif4.similar.html]

motif4

## Information for motif4

A
T
C
G
G
A
C
T
T
A
G
C
T
C
G
A
A
G
C
T
G
C
A
T
G
C
A
T
G
A
T
C
  
Reverse Opposite:  

C
T
A
G
C
G
T
A
C
G
T
A
T
C
G
A
A
G
C
T
A
T
C
G
C
T
G
A
T
A
G
C
  

|  |  |
| --- | --- |
| p-value: | 1e-451 |
| log p-value: | -1.039e+03 |
| Information Content per bp: | 1.638 |
| Number of Target Sequences with motif | 3697.0 |
| Percentage of Target Sequences with motif | 29.53% |
| Number of Background Sequences with motif | 4945.7 |
| Percentage of Background Sequences with motif | 13.80% |
| Average Position of motif in Targets | 101.1 +/- 53.4bp |
| Average Position of motif in Background | 100.2 +/- 66.5bp |
| Strand Bias (log2 ratio + to - strand density) | 0.1 |
| Multiplicity (# of sites on avg that occur together) | 1.16 |
| Motif File: | file (matrix) reverse opposite |

### Similar de novo motifs found

|  |  |  |  |  |  |  |  |
| --- | --- | --- | --- | --- | --- | --- | --- |
| Rank | Match Score | Redundant Motif | P-value | log P-value | % of Targets | % of Background | Motif file |
| 1 | 0.736 | G A T C C G A T A T C G G C A T A C G T C G T A G C A T A T C G G C T A A G T C | 1e-143 | -330.794375 | 12.67% | 6.39% | motif file (matrix) |
| 2 | 0.609 | C A G T T G C A C G A T A T C G G C T A G T A C C T A G A C G T T A C G C T G A | 1e-11 | -26.208438 | 1.35% | 0.76% | motif file (matrix) |
